# Activation of Phosphotyrosine-Mediated Signaling Pathways in the Cortex and Spinal Cord of SOD1^G93A^, a Mouse Model of Familial Amyotrophic Lateral Sclerosis

**DOI:** 10.1155/2018/2430193

**Published:** 2018-08-05

**Authors:** Cinzia Mallozzi, Alida Spalloni, Patrizia Longone, Maria Rosaria Domenici

**Affiliations:** ^1^Department of Neuroscience, Istituto Superiore di Sanità, Viale Regina Elena 299, 00161 Rome, Italy; ^2^Molecular Neurobiology Unit, Experimental Neurology, Santa Lucia Foundation, Via Ardeatina 306/354, 00142 Rome, Italy; ^3^National Center for Drug Research and Evaluation, Istituto Superiore di Sanità, Viale Regina Elena 299, 00161 Rome, Italy

## Abstract

Degeneration of cortical and spinal motor neurons is the typical feature of amyotrophic lateral sclerosis (ALS), a progressive neurodegenerative disease for which a pathogenetic role for the Cu/Zn superoxide dismutase (SOD1) has been demonstrated. Mice overexpressing a mutated form of the SOD1 gene (SOD1^G93A^) develop a syndrome that closely resembles the human disease. The SOD1 mutations confer to this enzyme a “gain-of-function,” leading to increased production of reactive oxygen species. Several oxidants induce tyrosine phosphorylation through direct stimulation of kinases and/or phosphatases. In this study, we analyzed the activities of src and fyn tyrosine kinases and of protein tyrosine phosphatases in synaptosomal fractions prepared from the motor cortex and spinal cord of transgenic mice expressing SOD1^G93A^. We found that (i) protein phosphotyrosine level is increased, (ii) src and fyn activities are upregulated, and (iii) the activity of tyrosine phosphatases, including the striatal-enriched tyrosine phosphatase (STEP), is significantly decreased. Moreover, the NMDA receptor (NMDAR) subunit GluN2B tyrosine phosphorylation was upregulated in SOD1^G93A^. Tyrosine phosphorylation of GluN2B subunits regulates the NMDAR function and the recruitment of downstream signaling molecules. Indeed, we found that proline-rich tyrosine kinase 2 (Pyk2) and ERK1/2 kinase are upregulated in SOD1^G93A^ mice. These results point out an involvement of tyrosine kinases and phosphatases in the pathogenesis of ALS.

## 1. Introduction

Amyotrophic lateral sclerosis (ALS) is a fatal neurodegenerative disease characterized by a progressive loss of motor neurons in the cortex, brain stem, and spinal cord [1]. The degeneration of motor neurons leads to skeletal muscle weakness, paralysis, and eventually death, with a mean survival between three and five years after disease onset [2–5]. Despite that the disease has been described since more than a century ago, the exact aetiology is still unknown. About 5–10% of ALS cases are inherited and among those about 20% are associated with missense mutations in the ALS1 locus on chromosome 21, which codes for Cu/Zn superoxide dismutase (SOD1) [6]. As familial and sporadic ALS (fALS and sALS, resp.) are symptomatically indistinguishable, it is likely that they share common pathogenetic mechanisms. Such mechanisms include protein misfolding, inflammation, oxidative stress, and mitochondrial dysfunction (reviewed in [7, 8]). Additionally, there is evidence for the involvement of excitotoxicity, as illustrated by the therapeutic effect of riluzole, a drug that blocks glutamatergic neurotransmission, which is currently the only disease-modifying drug available [9, 10]. Excitotoxicity is defined as an excessive activation of glutamate receptors by excitatory amino acids, such as glutamate, that initiates a series of cytoplasmic and nuclear processes promoting neuronal cell death [11, 12]. Overstimulation of the ionotropic glutamate receptors in neurons causes massive influx of calcium into the cytosol, and numerous enzymes are activated in response to the increase in intracellular calcium [13–15].

Several lines of evidence reported that src family protein tyrosine kinases are involved in excitotoxicity [16–19], pointing to their possible role in the pathogenesis of ALS.

In the present study, we analyzed the activities of src and fyn tyrosine kinases and of protein tyrosine phosphatases (PTP), with particular interest to the striatal-enriched protein tyrosine phosphatase (STEP), in synaptosomal fractions prepared from the motor cortex and spinal cord of a transgenic mouse model of ALS (G93A mice), expressing wild-type human SOD1 (SOD1^WT^) or overexpressing human mutant SOD1 (SOD1^G93A^). We found that mutation in SOD1 deeply modulates the activity of tyrosine kinases and phosphatases, influencing the phosphorylation state of several substrates, including NMDA receptor (NMDAR) subunit GluN2B, proline-rich tyrosine kinase 2 (Pyk2), and ERK1/2 kinase.

## 2. Materials and Methods

### 2.1. Mice

Adult B6.Cg-Tg(SOD1-G93A)1Gur/J mice expressing high copy number of mutant human SOD1 with a Gly93Ala substitution (SOD1^G93A^) and adult B6SJL-TgN(SOD1)2Gur mice expressing wild-type human SOD1 were originally obtained from Jackson Laboratories (Bar Harbor, ME, USA). For the mSOD1^G93A^ transgene, the G1 line was used, while for the SOD1^WT^, the N1029 line was used [20]. The two lines express a different number of copies, with the G1 having more than double the number of gene copies and the N1029 line expressing a comparable number of human SOD1 copies or even greater in the brain [20, 21]. Both lines are maintained and selectively bred in the hemizygous state on an F1 hybrid C57BL6 × SJL genetic background in the animal facility of the Fondazione Santa Lucia (Rome, Italy) by crossbreeding transgenic hemizygous males with C57BL/6 females. The transgenic progeny was genotyped by analyzing tissue extracts from tail tips as previously described [22]. The animals were kept under standardized temperature, humidity, and lighting conditions, with free access to water and food (standard pellets). Animal care and use followed the European Directive 2010/63/EU adopted by the Council of the European Union for animal experiments, and adequate measures were taken to minimize pain or discomfort. The experimental protocol was approved by the Italian Ministry of Health. The mice used in the present study develop degeneration in lower motor neurons and paralysis at about 120 days. The groups were transgenic human wild-type SOD1 mice (SOD1^WT^), transgenic G93A mice (SOD1^G93A^), and the age-matched nontransgenic controls. The mice used in the study were all male and all at 120 days of age. Before proceeding to the dissection of tissues for biochemical analyses, mice were deeply anesthetized and then sacrificed by decapitation. The brain, carefully removed, was washed in ice-cold phosphate-buffered saline, and a 1 mm coronal slice containing the motor cortex was dissected using a 1 mm coronal mouse Jacobowitz brain slicer (Zivic Miller). The slice was then carefully laid down on a glass slide under a dissection microscope, and the motor cortex M1 region was dissected as previously described [14]. Whole spinal cords were ejected from the vertebral column by means of sterile 0.1 M phosphate-buffered saline injection in the vertebral column. Tissues were immediately frozen on dry ice and stored at −80°C until use.

### 2.2. Materials

We used the following antibodies: polyclonal anti-STEP, anti-Pyk2 (pY402), anti-ERK1/2 (pT202/pY204), and anti-ERK1/2 from Cell Signaling Technology (Danvers, MA, USA); polyclonal anti-Pyk2, monoclonal anti-*β*-actin, and polyclonal anti-fyn from Santa Cruz Biotechnology (Santa Cruz, CA, USA); monoclonal anti-v-src (Ab1, clone 327) from Calbiochem (EMD Chemical, Merck, Darmstadt, Germany); polyclonal anti-GluN2B (pY1472), anti-GluN2B, and monoclonal anti-phosphotyrosine (pY, clone 4G10) from Millipore Bioscience Research Reagent (Billerica, MA, USA); and peroxidase-conjugated goat anti-mouse and goat anti-rabbit from Bio-Rad (Hercules, CA, USA). Protein A/G PLUS agarose was from Santa Cruz Biotechnology and Trysacryl-immobilized protein A from Thermo Scientific (Waltham, MA, USA). Nitrocellulose was from Schleicher and Schuell Bioscience Inc. (Dassel, Germany); *p*-nitrophenyl phosphate (*p*-NPP) and enolase were from Sigma Chemical (St. Louis, MO, USA). Complete protease inhibitor cocktail was from Roche Diagnostics (Basel, Switzerland). [*γ*^32^P] ATP (>3000 Ci/mmol) was obtained from DuPont NEN (Boston, MA, USA).

### 2.3. Synaptosome Preparation

Crude synaptosomal fraction was prepared from the motor cortex and spinal cord according to a previous report [23]. Briefly, brain tissues were homogenized in 10 vol (*w*/*v*) of ice-cold buffer A (0.32 M sucrose, 5 mM Hepes-NaOH (pH 7.4), 0.5 mM EGTA, 5 mM NaF, and 1 mM Na_3_VO_4_) in the presence of protease inhibitor mixture (Complete; Roche Molecular Biochemicals, Indianapolis, IN, USA) using a Teflon-glass grinder. The homogenate was centrifuged at 1000 ×g for 5 min at 4°C; the resulting pellet (P1), containing nuclei and debris, was discarded whereas the supernatant (S1) was collected and centrifuged at 9200 ×g for 15 min. The supernatant (S2) was removed, and the pellet was washed in homogenization buffer and centrifuged at 10200 ×g for 15 min at 4°C to obtain a crude synaptosomal fraction (P2). The purity of synaptosome was previously evaluated by Western blotting using the markers specific to synaptosome [14]. The samples prepared for determination of PTP and STEP activity were solubilized in buffer A without phosphatase inhibitors. Protein content was determined by bicinchoninic acid assay (BCA kit, Thermo Scientific, Waltham, MA, USA), and synaptosomes were diluted to a concentration of 1 mg/ml.

### 2.4. Immunoprecipitation and *In Vitro* Kinase Assay

Immunoprecipitation of src and fyn kinases and *in vitro* kinase assay were performed as previously described [24]. Synaptosomes were solubilized by incubation for 1 hour at 0°C with an equal volume of 4X RIPA buffer (100 mM Tris–HCl (pH 7.5), 0.6 M NaCl, 4% (*w*/*v*) Triton X-100, 4% (*v*/*v*) Na-deoxycholate, 0.4% (*v*/*v*) SDS, 0.4 mM Na_3_VO_4_, 20 *μ*g/ml leupeptin, 20 *μ*g/ml aprotinin, and 4 mM PMSF), diluted twice with TBS (50 mM Tris–HCl (pH 7.4) and 150 mM NaCl), and then centrifuged at 12000 ×g for 15 min at 4°C. After centrifugation, the supernatant was incubated with 25 *μ*l of 50% (wt/vol) protein A/G PLUS agarose beads (Santa Cruz) for 1 hour at 4°C, clarified by centrifugation, and incubated overnight at 4°C in a rotating wheel with the different antibodies (2 *μ*g/ml monoclonal anti-v-src and 2 *μ*g/ml polyclonal anti-fyn). Src and fyn immunocomplexes were precipitated by the addition of 50% (wt/vol) protein G or protein A beads, respectively, and incubated at room temperature for 1 hour under gentle rotation. The beads were collected by centrifugation and washed twice with 1X RIPA buffer, twice with TBS, and once with kinase buffer (25 mM Tris–HCl (pH 7.5), 10 mM MnCl_2_, and 0.1 mM Na_3_VO_4_). The kinase reaction was carried out in 20 *μ*l of kinase buffer containing 1 *μ*g of enolase and 1 *μ*Ci of [*γ*^32^P] ATP (>3000 Ci/mmol) at room temperature for 10 min. The reaction was stopped by adding 10 *μ*l of 4X loading buffer, and the samples were subjected to Western blot. The gels were dried and exposed to X-ray film for autoradiography. Dried gels were used for direct determination of radioactivity using a phosphor imager instrument (Packard, Canberra, CO).

### 2.5. Western Blot Analysis

Samples prepared for Western blot analysis were solubilized in 4X loading buffer, boiled for 5 min, and proteins were resolved on 10% SDS-PAGE. Proteins were transferred to nitrocellulose paper at 35 V overnight. Blots were washed with TBS-0.05% Tween 20 (TTBS) and blocked with 3% BSA in TTBS for 2 hours. Washed nitrocellulose filters were incubated overnight at 4°C with the appropriate antibody. After extensive washes in TTBS, the immunoreactive bands were detected by chemiluminescence coupled to peroxidase activity (ECL), according to the manufacturer's specifications (ECL Kit, Thermo Scientific, Waltham, MA, USA), and quantified using a Bio-Rad ChemiDoc XRS system.

### 2.6. PTP and STEP Activity

Total PTP activity was detected in synaptosomes using *para*-nitrophenyl phosphate (*p*-NPP) as substrate, according to the procedure previously described [25]. Briefly, synaptosomes were suspended in assay buffer (25 mM Hepes (pH 7.4), 20 mM MgCl_2_, and 0.1 mM PMSF) containing 15 mM *p*-NPP and incubated at 37°C for 30 min. The reaction was stopped by the addition of 0.1 mM NaOH. Samples were centrifuged, and the release of *p*-nitrophenol from *p*-NPP was measured in the supernatant at 405 nm.

The activity of STEP was measured in the immunocomplexes obtained from synaptosomes as described above using a polyclonal anti-STEP antibody. STEP immunocomplexes were precipitated by the addition of 50% (*w*/*v*) Trysacryl-immobilized protein A beads. To measure the activity of STEP, the immunoprecipitates were suspended in 200 *μ*l of assay buffer containing 15 mM *p*-NPP and incubated for 60 min at 30°C under gentle stir. The activity was determined in the clarified supernatants by measuring the absorbance at 405 nm of *p*-nitrophenol.

### 2.7. Statistical Analysis

All data are presented as mean ± SEM. The Mann–Whitney *U* test or Student's *t*-test was used for single comparisons. Differences among multiple groups were analyzed by the Kruskal-Wallis nonparametric analysis of variance (followed by Dunn's test for multiple comparisons). A *p* value ≤ 0.05 indicated statistically significant differences.

## 3. Results

### 3.1. Tyrosine Phosphorylation Signal Is Enhanced in the Motor Cortex and Spinal Cord of SOD1^G93A^ Mice

**Figure 1:**
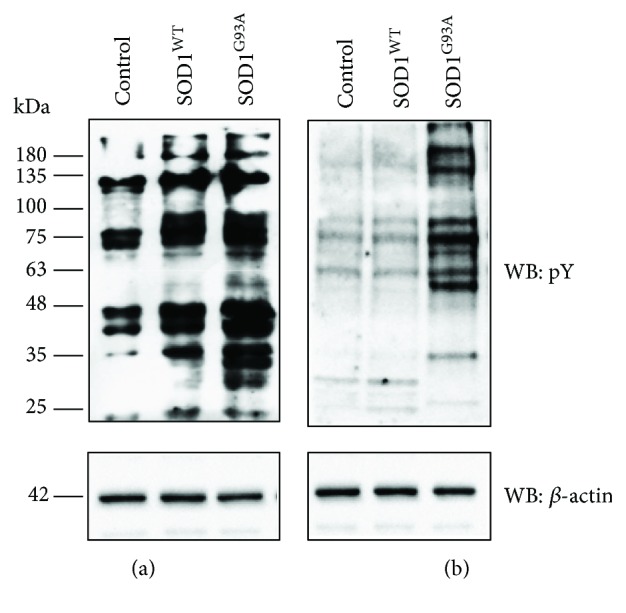
Activation of phosphotyrosine signal in the cortex and spinal cord of SOD1^G93A^ mice. Synaptosomes (1 mg/ml) were prepared from the motor cortex (a) and spinal cord (b) of control, SOD1^WT^, and SOD1^G93A^ animals, and phosphotyrosine content was evaluated in solubilized synaptosomes by Western blot (WB) analysis using an anti-pY antibody. The nitrocellulose was also probed with an anti-*β*-actin antibody to evaluate the amount of loaded proteins (lower panels). Immunoreactive bands were detected by ECL. Data are representative of four separate experiments.

The pattern of phosphotyrosine distribution was assayed by Western blot analysis using an anti-phosphotyrosine (pY) antibody in synaptosomes prepared from the motor cortex and spinal cord of control, SOD1^WT^, and SOD1^G93A^ mice. Synaptosomes retain the elaborated structural specialization of isolated nerve terminals [26] and are a particularly useful model to explore specific proteins modified by physiological oxidants. As shown in Figure 1, tyrosine-phosphorylated proteins increased in SOD1^G93A^ mice, both in the cortex and spinal cord (a and b, resp.). A slight increase was also observed in the SOD1^WT^ cortex when compared to control mice (Figure 1(a)).

### 3.2. Activation of Src and Fyn Tyrosine Kinases in the Cortex and Spinal Cord of SOD1^G93A^ Mice

**Figure 2:**
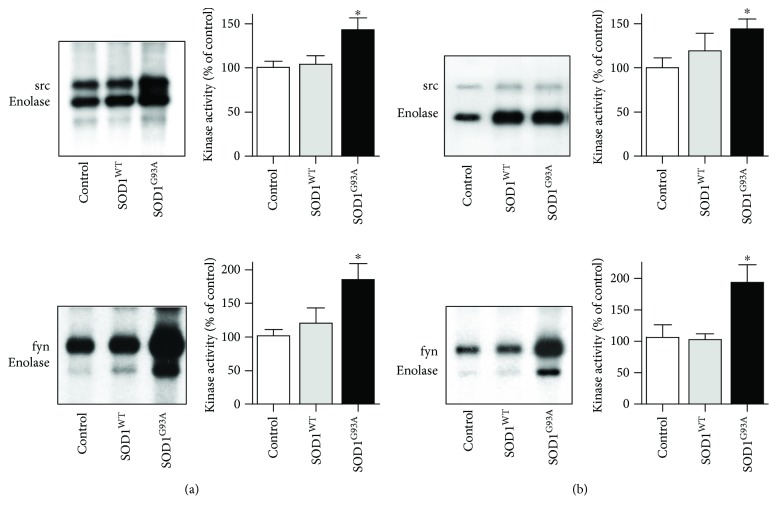
The activity of src and fyn kinases is upregulated in the cortex and spinal cord of SOD1^G93A^ mice. *In vitro* kinase activity of src (upper panels) and fyn (lower panels) isolated from the cortex (a) and spinal cord (b) obtained from control, SOD1^WT^, and SOD1^G93A^ mice. Enolase was used as an exogenous substrate. The [^32^P]-labelled proteins were revealed on dried gel by exposure to X-ray film, and the extent of [^32^P] incorporation in the substrate enolase was quantified using phosphor imager instrument and expressed as percentage of the value of the control samples (100%). The bar graphs represent the means ± SEM of four independent experiments. ^∗^Significantly different from SOD1^WT^ and control (*p* ≤ 0.05, Kruskal-Wallis followed by Dunn's test).

The results described above prompted us to investigate the contribution of specific tyrosine kinases and PTP to the modulation of the phosphotyrosine signal. To this end, we performed *in vitro* kinase assays on src family tyrosine kinases src and fyn, immunoprecipitated from synaptosomes of the cortex and spinal cord. We measured the kinase activity as autophosphorylation and phosphorylation of the exogenous substrate enolase. As shown in Figure 2, src and fyn activities were upregulated in SOD1^G93A^ compared with control and SOD1^WT^ mice, both in the motor cortex (Figure 2(a)) and in the spinal cord (Figure 2(b)). When the kinase activities were expressed as percentage variation of the respective controls, a statistically significant increase in the activity of both kinases was evident in the motor cortex and spinal cord of SOD1^G93A^ with respect to control and SOD1^WT^ mice (Figure 2).

### 3.3. Downregulation of Tyrosine Phosphatase Activity in the Cortex and Spinal Cord of SOD1^G93A^ Mice

**Figure 3:**
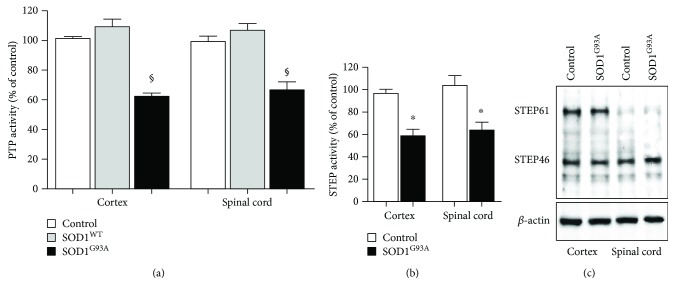
Effects of G93A mutation in SOD1 mice on PTP and STEP activities. (a) PTP activity was measured in synaptosomes obtained from the cortex and spinal cord of control, SOD1^WT^, and SOD1^G93A^ animals. The activity is expressed as percentage variation of control values. The bar graphs represent the means ± SEM of five independent experiments for each group. ^§^Significantly different from SOD1^WT^ and control (*p* < 0.05, Kruskal-Wallis followed by Dunn's test). (b) STEP protein was immunoprecipitated by a specific polyclonal antibody from solubilized synaptosomes prepared from the cortex and spinal cord of SOD1^G93A^ and control mice. The phosphatase activity of the STEP-immunocomplex is expressed as percentage variation of control values (100%). The bar graphs represent the means ± SEM of four independent preparations. ^∗^Significantly different from control (*p* < 0.05, Mann–Whitney *U* test). (c) Western blot analysis with an anti-STEP polyclonal antibody of solubilized synaptosomes prepared from the cortex and spinal cord of control and SOD1^G93A^ mice. The nitrocellulose was also probed with an anti-*β*-actin antibody to evaluate the amount of loaded proteins (lower panel). The immunoreactive bands were detected by ECL. The results shown are representative of four independent experiments.

We next evaluated the contribution of PTPs. The phosphatase assay revealed a decrease in enzymatic activity of total PTP in SOD1^G93A^ synaptosomes, both in the cortex and spinal cord. When PTP activity was expressed as percentage variation of controls, a statistically significant reduction was observed in the cortex and spinal cord of SOD1^G93A^ with respect to controls and SOD1^WT^ (Figure 3(a)).

Among the brain-specific tyrosine phosphatases that could be modulated by the mutation of SOD1, we focused on STEP. To measure the activity of STEP, we immunoprecipitated the protein by a specific antibody and evaluated the phosphatase activity associated with the immunocomplex. As shown in Figure 3(b), STEP activity was downregulated in SOD1^G93A^, both in the cortex and in the spinal cord (58.7 ± 5.9% and 63.7 ± 7.4% of reduction, resp., *p* < 0.05 with respect to controls, Mann–Whitney *U* test). Western blot analysis revealed that the two major isoforms, STEP61 and STEP46, although differently expressed in the cortex and spinal cord (STEP61 is less expressed in the spinal cord than in the cortex), did not differ between control and SOD1^G93A^ mice (Figure 3(c)).

### 3.4. Tyrosine Phosphorylation of GluN2B, ERK1/2, and Pyk2

**Figure 4:**
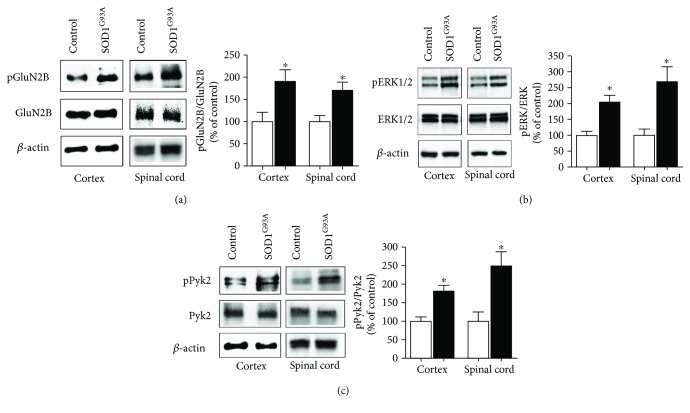
Tyrosine phosphorylation of STEP substrates. The tyrosine phosphorylation levels of GluN2B (a), ERK1/2 (b), and Pyk2 (c) were evaluated by Western blot analysis using specific antibodies that recognized the phosphorylated and nonphosphorylated forms of each enzyme in the cortex and spinal cord of control and SOD1^G93A^ animals. Anti-*β*-actin antibody was used to evaluate the amount of loaded proteins. The immunoreactive bands were detected by ECL. The immunoblots are representative of three independent experiments. The bar graphs represent quantification by densitometric analysis of band intensity relative to the appropriate nonphosphorylated proteins, expressed as percentage of the relative controls. White and black columns: control and SOD1^G93A^ mice. The bar graph represents the means ± SEM of 3 independent experiments. ^∗^Significantly different from control (*p* ≤ 0.05, Student's *t*-test).

In order to verify whether the inhibition of STEP activity observed in SOD1^G93A^ mice resulted in the hyperphosphorylation of tyrosine residues of its substrates, we evaluated the phosphorylation status of the GluN2B subunit of the NMDAR, Pyk2, and ERK1/2, three known STEP substrates [27–29]. We found by Western blot analysis that in the presence of a decreased activity of STEP, as in SOD1^G93A^ mice, GluN2B tyrosine phosphorylation level was significantly upregulated (Figure 4(a)) as well as the phosphorylation level of ERK1/2 and Pyk2 (Figures 4(b) and 4(c), resp.), both in the cortex and in the spinal cord of SOD1^G93A^ mice.

## 4. Discussion

In the present study, we highlight the impact of phosphotyrosine-mediated signaling in the pathogenesis of ALS. In mice overexpressing the mutant human SOD1 (SOD1^G93A^), we demonstrate, both in the motor cortex and spinal cord, that (i) the protein-associated tyrosine phosphorylation signal is increased, (ii) the enzymatic activity of two members of the src kinase family, src and fyn, is upregulated, and (iii) the activity of PTP, in particular of STEP, is inhibited. Moreover, we found that the tyrosine phosphorylation of the NMDAR subunit GluN2B, the major tyrosine phosphorylated substrate in the brain, and that of Pyk2 and ERK1/2 are greatly amplified.

SOD1^G93A^ mice develop a syndrome that closely resembles the human disease, but the molecular pathways that cause motor neurodegeneration are still largely debated. The mutations of SOD1 confer to the enzyme a “gain-of-function,” leading to increased hydrogen peroxide levels and reactive oxygen species (ROS) [30]. In ALS, various indices of ROS-induced damage have been reported within the specific brain region that undergoes selective neurodegeneration [31]. In addition, oxidative stress and the formation of oxygen free radicals are key components of the glutamate-induced neurotoxicity, a pathogenetic mechanism that has received much attention as a critical player in ALS development and progression [32]. Several lines of evidence reported that src family protein tyrosine kinases are involved in excitotoxicity [16–19], pointing to a possible role in the pathogenesis of ALS. Accordingly, we report the activation of two members of the src family kinases highly expressed in the central nervous system, src and fyn, both in the cortex and in the spinal cord of SOD1^G93A^ mice. We previously demonstrated that several oxidants are able to activate the src family tyrosine kinases, which are directly implicated in tyrosine phosphorylation of the NMDAR [33–35]. Indeed, NMDARs are particularly vulnerable to the action of free radicals, such as nitric oxide and superoxide anion, which can modulate the activity of tyrosine kinases and phosphatases and then control the functionality of NMDAR [33–38]. In fact, NMDAR activity is governed by a balance between tyrosine phosphorylation and dephosphorylation: the activation of src kinases and/or inhibition of phosphatase activity results in the enhancement of NMDAR function [39, 40]. The NMDAR subunits GluN2A and GluN2B are tyrosine phosphorylated and, in particular, the GluN2B subunit is the main tyrosine-phosphorylated protein in the postsynaptic density, with Tyr1472 as probably the most phosphorylated site in the brain. Thus, the hyperphosphorylation of GluN2B and the consequent increase in NMDAR activity, sustained by ROS-activated src signaling, might represent a causal link between SOD1 mutation and the excitotoxic phenotype characterizing ALS.

**Figure 5:**
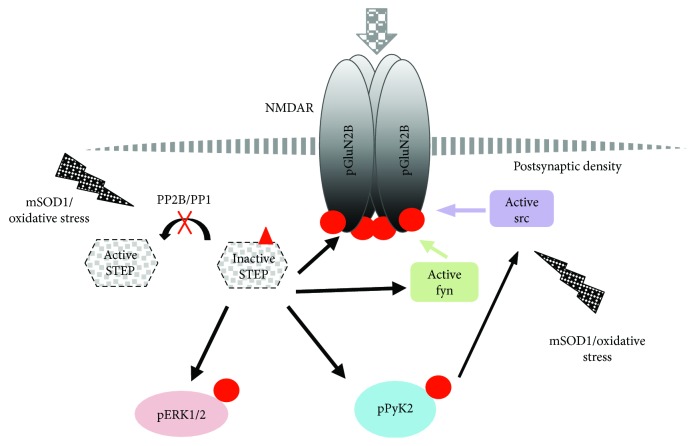
Schematic representation of STEP-mediated signaling cascade in ALS. Exposure to mutant SOD1 (mSOD1)/oxidative stress leads to inactivation of STEP, reasonably through inactivation of PP2B (calcineurin)/PP1 pathway, and activation of src kinases, both of which drive the tyrosine phosphorylation of the GluN2B subunit. In addition, as a consequence of STEP downregulation, Pyk2 becomes hyperphosphorylated and activated. Phospho-Pyk2 (pPyk2), in turn, upregulates NMDAR function by further activating src kinase, thus contributing to the potentiation of the activation loop. Red points and triangle represent phosphate groups at tyrosine and serine residues, respectively.

STEP is a tyrosine phosphatase highly expressed in the striatum which is also present in several other areas of the central nervous system, including the cerebral cortex and spinal cord [41]. STEP has two major isoforms, the membrane-associated STEP61 and the cytosolic STEP46 [42], differently expressed in the brain regions. STEP has been implicated in the pathophysiology of several neuropsychiatric diseases, and both high and low levels of STEP disrupt synaptic function and contribute to learning and behavioral deficits [43]. STEP, in its active form, dephosphorylates tyrosine residues on its substrates, causing their inactivation. In the case of glutamate receptor subunits, it dephosphorylates the GluN2B subunit of NMDARs at Tyr1472, counteracting the activity of src kinases, and promotes the internalization from surface membranes of the NMDARs, thus contributing to the homeostatic stabilization of the excitatory synapses [44–46]. In the spinal cord, the role for STEP61 in the modulation of nociception and in the development of inflammatory pain has been demonstrated [47, 48]. Li and collaborators [48] demonstrated that the hyperphosphorylation of GluN2B, fyn, and ERK2, induced by a reduction in the activity of STEP61, was critical to trigger pain hypersensitivity. Our study demonstrates for the first time a dysfunction of STEP activity in a mouse model of ALS and the hyperphosphorylation of its substrates, GluN2B, ERK1/2, and Pyk2. Pyk2 is a member of the focal adhesion kinase family, upstream of src in the signaling cascade through which tyrosine phosphorylation enhances the function of NMDAR [49]. Our results suggest that the reduced STEP activity resulted in the hyperphosphorylation of Pyk2, which may contribute to the potentiation of synaptic activity through activation of src kinase (Figure 5). We speculate that exposure to mSOD1/oxidative stress in SOD1^G93A^ leads to inactivation of STEP. Calcineurin is a phosphatase that regulates the activity of STEP through DARP32/PP1 cascade [41]. It has been recently demonstrated that calcineurin protein level and activity were significantly lower in the SOD1^G93A^ rat spinal cord [50]. In addition, decreased calcineurin enzyme activity in lymphocytes from ALS patients was reported [51]. Even though we did not evaluate calcineurin activity, it is reasonable to think that also in our SOD1^G93A^ mice, a reduced calcineurin activity fails to activate PP1, which in turn contributes to the maintenance of the phosphorylated/inactive status of STEP (Figure 5).

In contrast with previous papers demonstrating that STEP61 was the only isoform expressed in the dorsal spinal cord neurons [48, 52], we found that both isoforms were expressed in the spinal cord, with STEP46 even more expressed than STEP61 (Figure 3(c)). Although methodological differences (fetal versus adult spinal cord, dorsal versus total spinal cord) could be evoked, this discrepancy deserves further investigation.

The hyperphosphorylation of GluN2B at Tyr1472 correlates well with the role played by GluN2B in triggering NMDAR hyperfunctioning during inflammatory pain [53] and could be involved in the mechanisms of neuroinflammation that contributes to motor neuron degeneration in ALS [54, 55]. Interestingly, masitinib, a highly selective tyrosine kinase inhibitor, currently in phase 3 clinical development in ALS patients, modulates the neuroinflammation in the SOD1^G93A^ rat model of ALS and reduces the proliferation, migration, and inflammatory transcriptional profile in microglial cell cultures [56]. The hypothesis that tyrosine kinases could be involved in the pathogenesis of ALS was already suggested by the study of Jiang and collaborators [57] who demonstrated a 4.4-fold increase in the mRNA for c-Abl, a ubiquitous nonreceptor tyrosine kinase, in the motor neurons of sALS patients. Moreover, the c-Abl inhibitor dasatinib demonstrated neuroprotective properties *in vitro* and *in vivo* models of ALS [58]. Our findings are also supported by a recent paper of Imamura et al. [59] that demonstrates a protective role of bosutinib (src/c-Abl inhibitor) in motor neurons derived from iPSC of patients with familial or sporadic ALS. In addition, a tyrosine kinase inhibitor (saracatinib) has been demonstrated to reduce the downstream activation of Pyk2, leading to the restoration of synapse density and a gradual full recovery of behavioral deficits in transgenic mouse models of Alzheimer's disease [60], suggesting a broader exploitation of these drugs in the field of neurodegenerative diseases.

## 5. Conclusion

Our study demonstrates an increase in the phosphotyrosine-dependent signaling in the SOD1^G93A^ model of ALS and, in particular, identifies STEP as a new actor of the complex pathogenetic mechanisms of the disease. Whether this may help in finding new approaches for the treatment of this disease remains to be examined in depth.

## Data Availability

The datasets generated during the current study are not publicly available due to the lack of an institutional repository but are available from the corresponding author on reasonable request.
